# Performance Analysis of a Full-Scale Desalination Plant with Reverse Osmosis Membranes for Irrigation

**DOI:** 10.3390/membranes11100774

**Published:** 2021-10-11

**Authors:** Federico Leon, Alejandro Ramos

**Affiliations:** Departament of Process Engineering, University of Las Palmas de Gran Canaria, 35017 Las Palmas de Gran Canaria, Spain; alejandro.ramos@ulpgc.es

**Keywords:** seawater, reverse osmosis membranes, desalination, operating data, long-term

## Abstract

Reverse osmosis (RO) is the most widely used technology for seawater desalination purposes. The long-term operating data of full-scale plants is key to analyse their performance under real conditions. The studied seawater reverse osmosis (SWRO) desalination plant had a production capacity of 5000 m^3^/d for irrigation purposes. The operating data such as conductivities flows, and pressures were collected for around 27,000 h for 4 years. The plant had sand and cartridge filters without chemical dosing in the pre-treatment stage, a RO system with one stage, 56 pressure vessels, seven RO membrane elements per pressure vessel and a Pelton turbine as energy recovery device. The operating data allowed to calculate the average water and salt permeability coefficients (*A* and *B*) of the membrane as well as the specific energy consumption (*SEC*) along the operating period. The calculation of the average A in long-term operation allowed to fit the parameters of three different models used to predict the mentioned parameter. The results showed a 30% decrease of *A,* parameter *B* increase around 70%. The *SEC* was between 3.75 and 4.25 kWh/m^3^. The three models fitted quite well to the experimental data with standard deviations between 0.0011 and 0.0015.

## 1. Introduction

Desalination is the industrial process to remove salt from seawater or brackish water, obtaining desalinated water. 97.5% of the water that exists on our planet is salt water, 2.5% is fresh water and less than 1% of the latter is suitable for human consumption. Getting to make seawater drinkable is one of the possible solutions to the shortage of drinking water [1].

Seawater (SW) desalination in water treatment plants has evolved a lot in the last five decades, during which the desalination process and its technology have changed and become more and more profitable and efficient. Initially, the water desalination process was a thermal process that has been changing with the scientific technological advances towards a process by reverse osmosis, which dominates the current market [2,3,4,5].

Seawater desalination plants have produced potable water for many years to date, but the process has always been very expensive, both energetically, and economically. The origin of the energy necessary to produce water is mainly from the electrical systems that are isolated on islands, different on each island and on the mainland, which causes differences in the emission factor depending on its energy mix. The quality of the permeated water (boron rejection) is defined as an essential requirement for the water production that has a direct impact on the energy cost of the system [6,7,8].

Following the state of the art in water desalination and the evolution of this process, not only for a Canary regional level but also for a national and international level, there are different desalination procedures like multi-effect distillation (MSF), vapor compression (VC), multi-stage distillation (MED) and reverse osmosis (RO), which currently accounts for 65% of the total in the world [9,10,11,12,13,14,15].

The objective of this investigation is obtain improvements at desalination plants, based on the reduction of energy consumption in the production of fresh water. Consequently, reverse osmosis is the most suitable process due to the energy consumption to produce permeate water is lower than for other methods, so it occupies a privileged position in the sector. In fact, in the 21st century, research efforts in water desalination have focused on advances in reverse osmosis membranes, with higher surface area and lower energy consumption, as well as energy recovery systems to recover the brine pressure and to introduce it in the system reducing the energy consumption of the desalination process. The operation, maintenance and handling of the membranes has been studied in detail, due to their importance in energy savings, studying the optimization of the process to increase the energy efficiency [15,16,17,18,19].

Seawater desalination plants have produced potable water for many years to date, but the process has always been very expensive, energetically, and economically. The origin of the energy necessary to produce water is mainly from the electrical systems that are isolated on islands, different on each island and on the mainland, which causes differences in the emission factor depending on its energy mix. The quality of the permeated water (boron rejection) is defined as an essential requirement for the water production that has a direct impact on the energy cost of the system [20,21,22,23,24].

Regarding the production of desalinated seawater, for the specific case of SWRO plants in the Canary Islands, the following permeate flows can be confirmed: Gran Canaria (220,870 m^3^/d), Tenerife (106,034 m^3^/d), Fuerteventura (90,755 m^3^/d) and Lanzarote (87,480 m^3^/d). This represents a significant portion of the carbon footprint with respect to the overall footprint of each island, especially on Fuerteventura and Lanzarote. In this sense, renewable energies, mainly wind and solar photovoltaic, can make a great contribution. Therefore, the possibility of introducing renewable energies for the supply of electricity to the SWRO plants in Canaries is being studied to decrease the ecological and carbon footprints of the sector and also because of its considerable influence on the whole archipelago [25,26,27,28,29].

The long-term success of SWRO plants is due to the performance of the membranes loaded. Greater energy consumption is required in seawater desalination plants compared to of brackish water, for this reason the present study focuses on achieving energy consumption improvements in seawater desalination plants. The long-term operating data analysis is key to understand the viability of this process and to improve the performance of this kind of facilities [20,21,22,23,24,25,26,27,28,29]. The objective of this investigation is to study the operation data and to improve the energy consumption along 27,000 h over four years of operation of a SWRO plant in the Canaries used to produce water for irrigation [30,31,32,33,34].

The novelty of this work is based on how to maintain a good operation and performance for a long-term studying the operation data of a full-scale RO seawater desalination plant. BY following this long-term operating data, it was possible to study an RO desalination plant and to maintain the performance of the plant during four years without membrane replacement or chemical cleaning steps.

## 2. Materials and Methods

**Figure 1:**
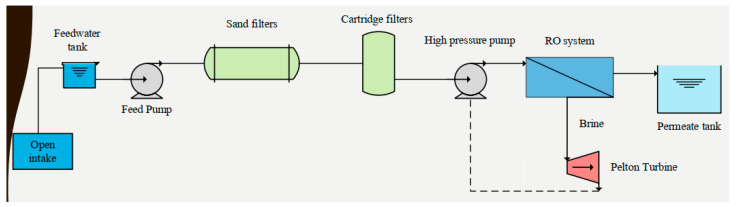
Desalination and plant diagram.

The SWRO plant (Figure 1) situated in Gran Canaria was designed to produce water for irrigation purposes. All the data of this installation during 27,000 h of operation for four years has been taken. The RO design is constituted by one bank with 56 pressure vessels. Each vessel has seven elements (Toray, city, state abbreviation if USA, country). Moreover, the system includes a Pelton turbine to recover energy.

The average water coefficient (A) and the salt permeability coefficient (B) are calculated using the operation data taken at this plant. It is thus possible to calculate the specific energy consumption (SEC) of the SWRO membranes during these four years. The calculation of the coefficient A permits to fit the parameters of three different models to get the commented parameter.

Figure 1 describes how the process is managed to get the appropriate high quality permeate water. There is an open intake with a feed water tank and pumping before physical pretreatment with sand filters in a first stage and cartridge filters in a second stage. No chemical pretreatment (not even an anti-scalant) is used due to the high quality of the feed water. After pretreatment, there is a high-pressure pump before the reverse osmosis system in one stage. The brine goes directly to the Pelton turbine and the product ends up in the potable water tank.

The operating conditions of any RO desalination plant such as pressure, flux recovery, and feedwater conditions can change, causing a variation in the product flow and the percentage of rejection. The average water and salt permeability coefficient were determined to evaluate the operation of the membranes installed which have the following technical specifications expressed in Table 1.

Greater energy consumption is required in seawater desalination plants compared to brackish water, and for this reason the present study focuses on achieving energy improvements in seawater desalination plants.

The carbon footprint is defined as the total amount of greenhouse gases emitted by direct or indirect effect of an individual, organization, event, or product. The energy mix is the distribution and weighting of the different energy sources (fossil energy, nuclear, renewable) necessary to respond to the needs of a demand determined. The ecological footprint is an index that refers to a specific demand of the nature of an organization or population. Their ecological footprint is the area of natural environment necessary for producing the resources they consume and absorb the waste they generate.

We are faced with the problem of membrane aging, which means an increase in the pressure and energy consumption, a reduction in quality and a decrease in the flow of permeated in time. It has been observed as a useful tool for improving energy efficiency the introduction of testing with high rejection and low consumption reverse osmosis membranes to reduce the costs.

## 3. Results

**Figure 2:**
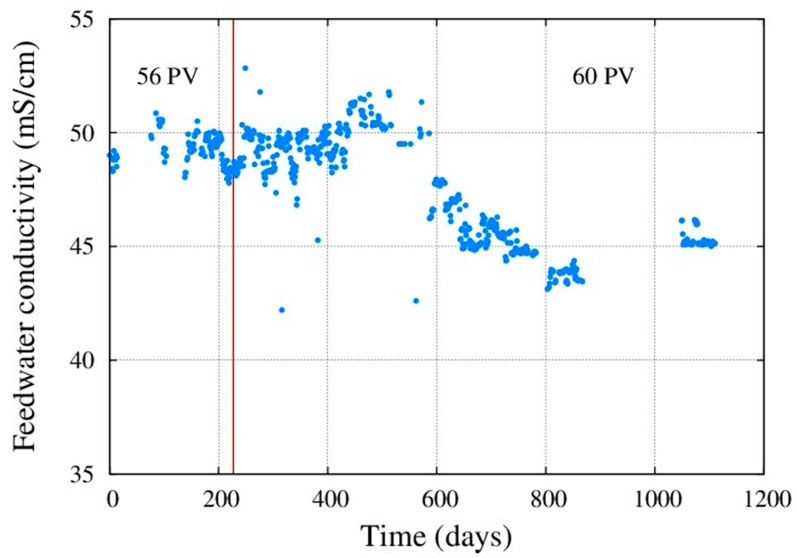
Feed conductivity.

The raw water conductivity varied during the seasons, but it never exceeded 55,000 μS/cm (Figure 2). Feed water inorganic composition is shown in Table 1.

**Figure 3:**
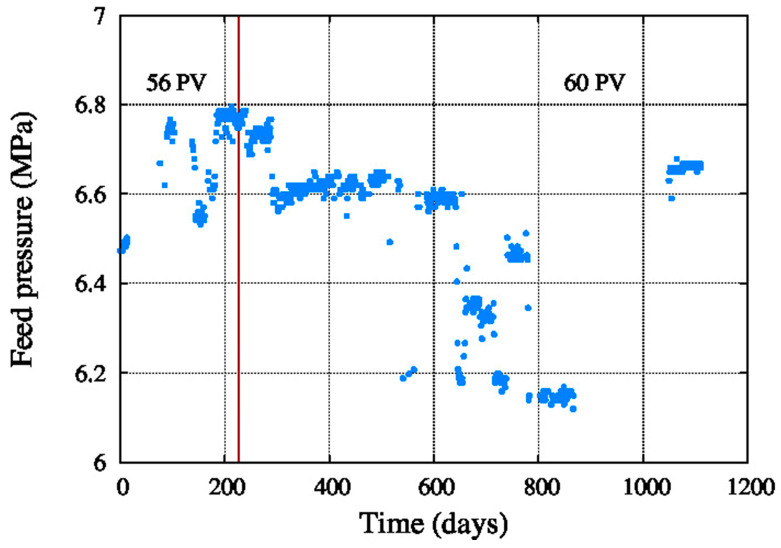
Feed pressure.

As shown in the Figure 3 the feed pressure was between 6.1 MPa and 6.8 Mpa. This was due to increased performance decay of the pressure vessels, specially fouling, scaling and compaction during these years.

The results show a certain dispersion of the data (especially related to those of feed pressure and correlation with the feed water conductivity). An increase of feed water conductivity will increase the feed water pressure and vice versa. Therefore, new resources would be required to continue studying this plant. This study tries to compare the performance of the membranes and to determine the optimal configuration.

According to the results, feed water conductivity decreases, and feed pressure decreases mainly after 600 days operation, both together. One can find bthe ion concentrations of the feed water in Table 2.

**Figure 4:**
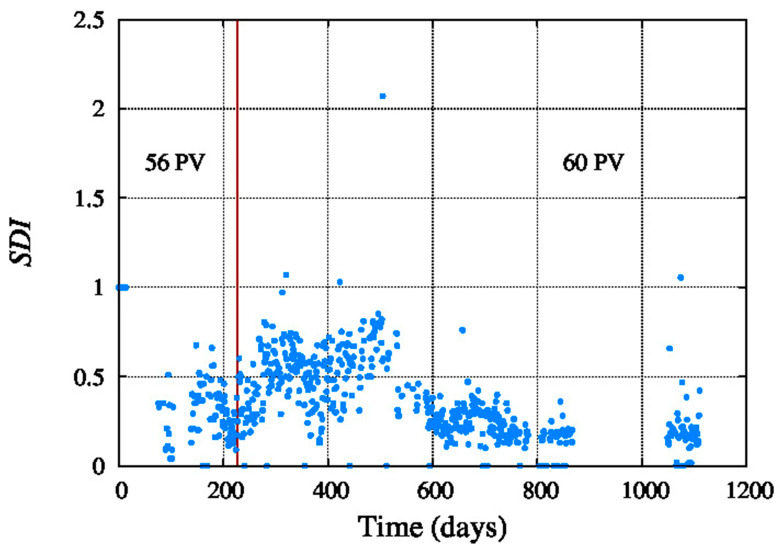
Feed SDI.

Figure 4 shows the feed SDI, which is mostly lower than 1 and on average below around 0.3. The low SDI, shown in the figure, indicate an opportunity to avoid a complicated chemical pre-treatment for this seawater reverse osmosis system. In fact, it is operating without any sodium metabisulphite, no sodium hypochlorite, no sulphuric acid and also no anti-scalant. According to the registered data this installation is operating well, within projected requirements, with good results for the client.

**Figure 5:**
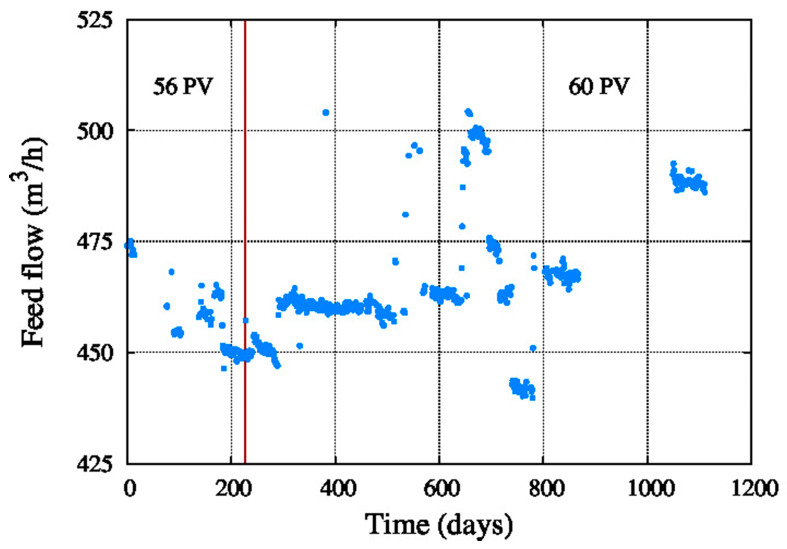
Feed flow.

Figure 5 shows the water feed flow, which was between 410 and 510 m**^3^**/h during the operating period. Therefore, the feed flow is stable in between this interval.

**Figure 6:**
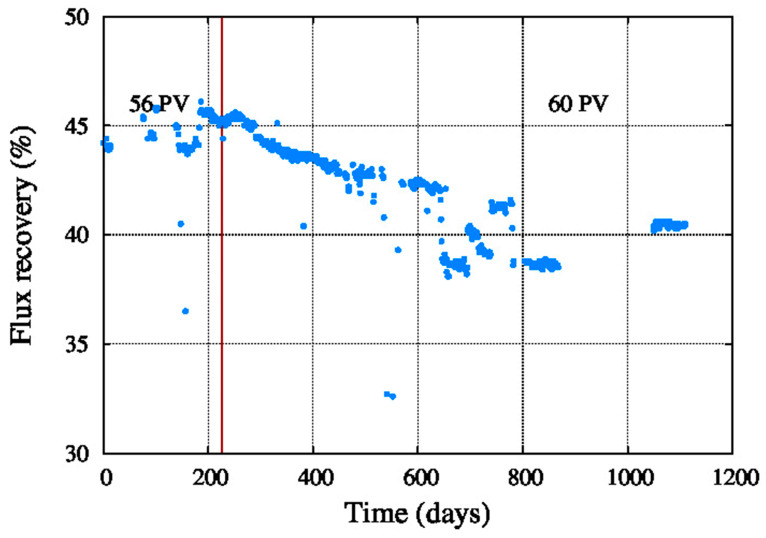
Recovery.

Figure 6 shows the decrease of the system recovery from 46% to 38% operating with 56 pressure vessels of seven elements each one. Therefore, after approximately 900 days of operation the number of pressure vessels was increased to 60 to boost the system recovery to at least 40%. This figure shows an increased performance decay which could be due to fouling, scaling and compaction occurred during the years of operation.

**Figure 7:**
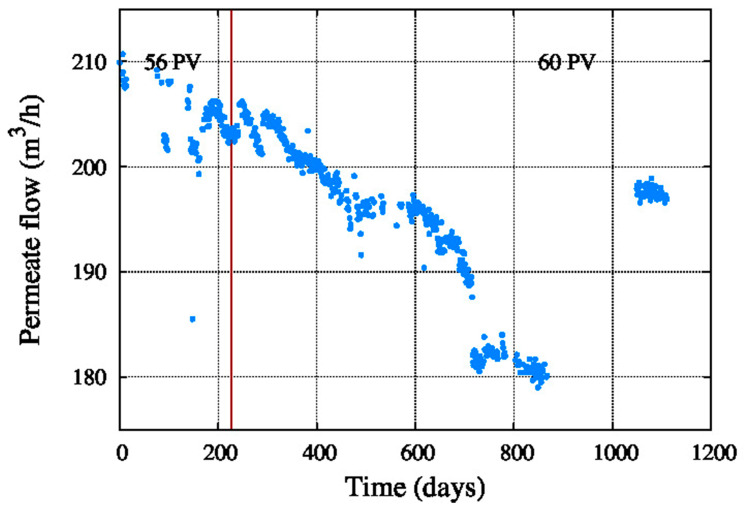
Permeate flow.

Figure 7 shows the decrease in the permeate flow during the operating period, due to the decrease of the recovery. This reduction of the permeate flow and the recovery could both be due to the fouling of the membranes, scaling and element compaction. Due to this the number of pressure vessels was increased from 56 to 60 to get more permeate flow. After 1000 days operation the permeate flow was increased again by introducing four more pressure vessels in the train.

**Figure 8:**
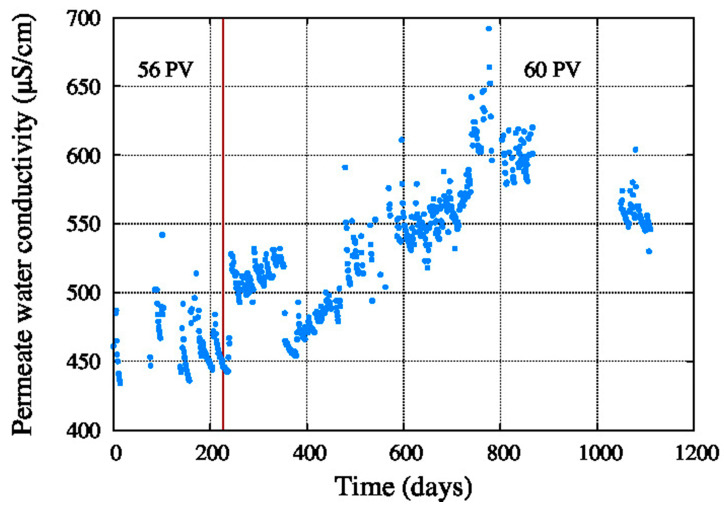
Permeate conductivity.

Figure 8 shows that the permeate water conductivity of the total RO system was in a range between 200 μS/cm and 800 μS/cm most of the time, which is quite acceptable for the mentioned irrigated crop. After the addition of 4 pressure vessels with new elements from Toray, the permeate water conductivity decreased and the permeate flow increased after 1000 days of operation.

**Figure 9:**
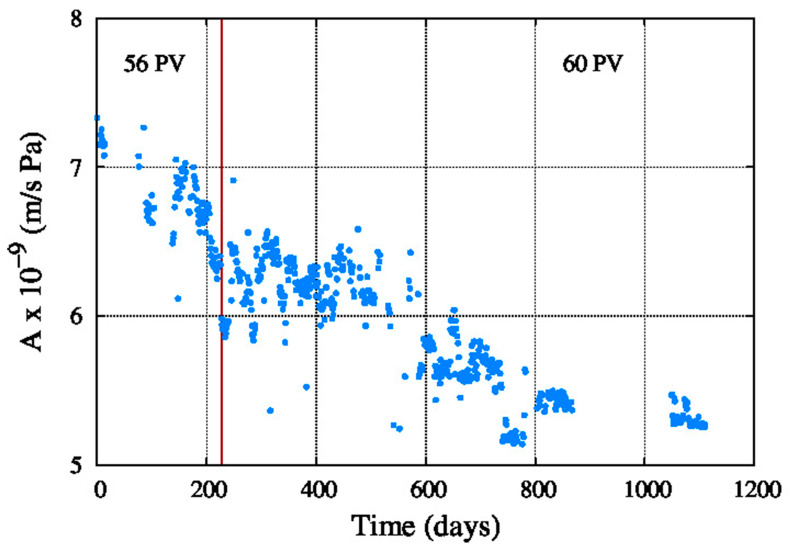
Average water permeability coefficient.

**Figure 10:**
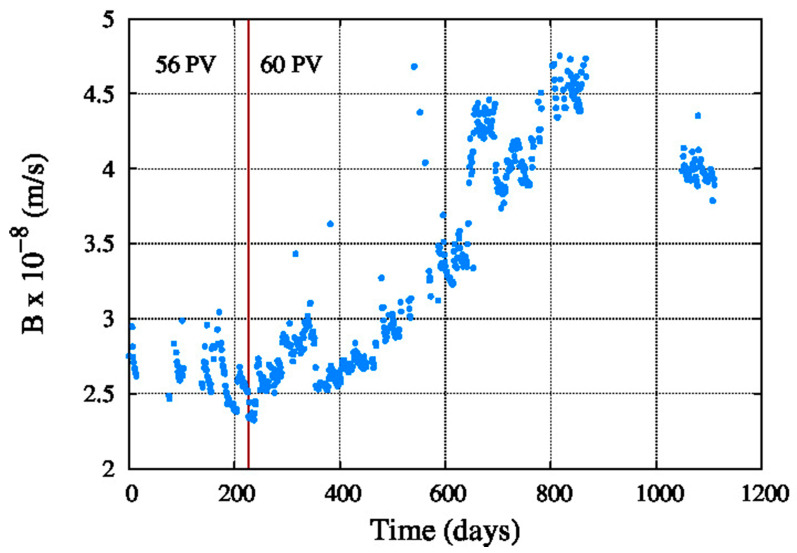
Average salt permeability coefficient.

Figure 9 shows the average water permeability coefficient, which decreased since the startup of the system until the end of the period. The same happens in Figure 10 with the average salt permeability coefficient, but in this case the value is increasing with the aging of the reverse osmosis membranes.

**Figure 11:**
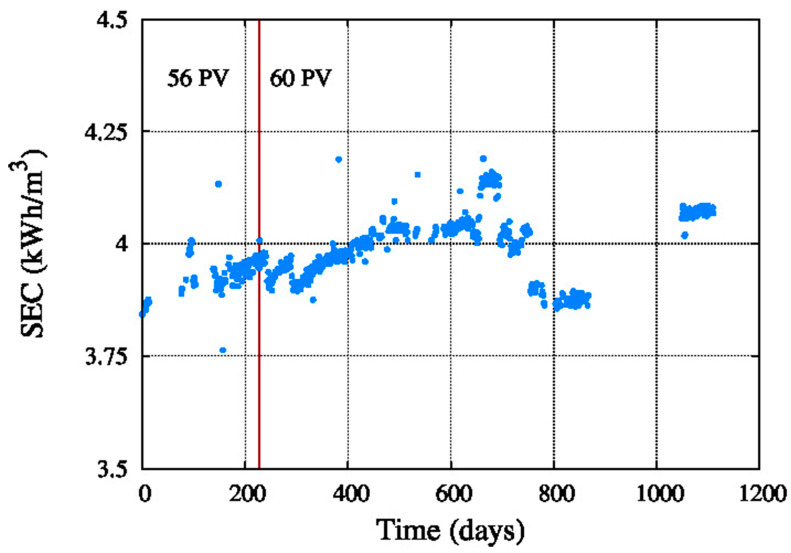
System energy consumption.

Figure 11 shows the energy consumption of the system, which is in between 3.75 kWh/m^3^ and 4.25 kWh/m^3^ of produced permeate water, with an average value around 4 kW/m^3^. Considering that the energy recovery device is a Pelton turbine these values are acceptable.

The energy consumption also depends on the feed pressure, which is in between 6.1 MPa and 6.8 MPa, so the difference is not too much, only 0.7 MPa, and the energy consumption is stable during the studied period of the system.

Finally, the cost of energy consumption in the pumps and mainly in the high-pressure pump is by far the most significant of a seawater desalination plant and we can reduce it considerably with the introduction of latest generation reverse osmosis membranes. In this case, the high-pressure pump consumption is about 3.04 kWh/m^3^. If we consider a price of 0.06 EUR/kWh the consumption will be 0.18 EUR/m^3^ of permeate water. In this plant with permeate flow of 5000 m^3^/d the cost of the energy consumption per day is 900 EUR.

It is possible to compare with other plants, for example with reference [5] and the following graphic for a seawater RO plant of 100,000 m^3^/d. In our case 900 EUR/day is for a production of 5000 m^3^/d so to produce 100,000 m^3^/d the cost should be 18,000 EUR/day which is lower than that of the referenced plant [5], thus demonstrating the better performance of the plant studied in this article.

Considering these parameters, for a typical production of a seawater plant of 100,000 m^3^/d capacity and our plant of 5000 m^3^/d adjusted to 100,000 m^3^/d, we obtain the following common results at an average temperature 22 °C for different scenarios (Table 3) [5].

## 4. Conclusions

The calculation of the average A in long-term operation allowed us to fit the parameters of three different models used to predict the mentioned parameter. The results showed a 30% decrease of A, while parameter B increased around 70%. The SEC was between 3.75 and 4.25 kWh/m^3^ which is a good value considering a Pelton turbine was used.

The introduction of more pressure vessels with new elements increased the permeate flow after 1000 days operation, restoring the permeate quality and energy consumption.

The models fitted could not be shown, but the three models fitted quite well to the experimental data with standard deviations between 0.0011 and 0.0015.

Using this long-term operating data, it has been possible to study an RO desalination plant and to improve the performance of the plant during 4 years without membrane replacement and chemical cleaning steps.

The long operation data will continue to be taken in the future to quickly follow up on any deviation and to act as soon as possible with any operation decision or even chemical cleaning to increase the good performance of the elements and the plant.

The future replacement will be studied as a partial replacement, only changing the first element of each vessel which is the most damaged and introducing the new one in the last position (on the brine side) to protect it.

## Figures and Tables

**Table 1:** Technical specifications of the RO membranes.

Feed Water	Seawater
Salt rejection	99.75%
Product Flow rate	16.00 m^3^/day

**Table 2:** Feed water inorganic composition.

Ion	Concentration mg/L
Ca^2+^	165
Mg^2+^	306
Na^+^	12,514
K^+^	642
HCO_3_^-^	488
SO_4_^2-^	1076
NO_3_^-^	308
Cl^-^	19,586
SiO_2_	20
TDS	35,313

**Table 3:** Plant comparison under different scenarios.

RO Plant (Age)	Pressure (bar)	Power (kW)	Energy (kWh/d)	Cost (€/d)
A (0 years)	66.6	10,023.5	240,564.9	21,625.6
B (1 year)	68.4	10,294.4	247,066.7	22,210.1
C (2 years)	69.6	10,475.0	251,401.2	22,599.7
D (3 years)	70.8	10,655.7	255,735.7	22,989.4
E (4 years)	72.0	10,836.3	260,070.2	23,379.0
F (5 years)	73.2	11,016.9	264,404.7	23,768.7
Our plant (4 years)	55.4	8343.1	200,233.3	18,000.0
